# Transformative community projects in East Germany's rural spaces: exploring more sustainable forms of learning, working, and living

**DOI:** 10.3389/fsoc.2023.1164293

**Published:** 2023-05-24

**Authors:** Joachim Broecher, Janet F. Painter

**Affiliations:** ^1^Department of Social and Emotional Learning, University of Flensburg, Flensburg, Germany; ^2^School of Education, Lenoir-Rhyne University, Hickory, NC, United States

**Keywords:** civil society, community project, unconditional basic income, self-directed learning, work-life balance, utopian thinking, social transformation

## Abstract

Increasingly people experience alienation in educational institutions, in work life, and fragmentation in their personal life. This study explores more self-determined, healthy, and sustainable forms of working, learning, and living through a dynamic process that began in 2020 with the purchase of an old homestead in Eastern Germany. Through the remodeling of the buildings and grounds, the first social and cultural references emerged. Along with practical uses, the farm project sees itself as a future workshop or think tank. The resulting consideration includes ideas of compulsory schooling woven into a self-designed format and the introduction of an unconditional basic income. These components could lead to thousands of such projects in rural and urban areas. Drawing from communitarianism, the belief is that an active civil society must take on social, economic, and educational responsibilities and offer children and young people improved conditions in which to grow up. Theory development on the individual components exists, such as entrepreneurship, transformation, community-building, basic income, or self-directed learning but not on the interaction of these variables in the overall context. We tentatively call this integrated design a *transformative community project*.

## Introduction

Examining decades of German society's development, one can acknowledge the existence of positive attributes such as stable state institutions, a robust public education and school system, a functioning welfare state system, and a sufficiently stable labor market. However, one realizes that there is also a shortage of skilled workers, for example, in the field of geriatric care or teaching within schools. Concurrently, signs of dissolution exist in various areas of society, disintegration of families, an unconnected coexistence of different ethnic communities, or lives that sometimes take place in escapist, virtual worlds. A common narrative or a value-based framework that could hold society together from within is scarce. The functionalized world of work yields human experiences that often lack professional self-realization. Competition, indifference, and alienation frame the work–life experience (e.g., Sayers, 2011; Shantz et al., 2014, 2015; Kalekin-Fishman and Langman, 2015; Bousquet, 2023). Parents leave their children at crèches, daycare centers, and schools, often for the workday. They rush to their 9–5 job, then retrieve their children and try to have nominal family time together before repeating the process. A primary goal of the education system is to prepare youth to cope with such a working world of jagged, disconnected living arrangements. Responsible authorities and ministries attempt to make school lessons better and more effective, through increased management, control, performance measurement, and support. Youngsters learn in neon-lit classrooms, often reduced to sedentary and book-focused learning experiences, far away from forests, meadows, and fields. However, complex difficulties increasingly appear among youth. When adjoined with conditions of instability, including broken family relationships and complex migration backgrounds, unresolved trauma and uprooting experiences, educational careers, and successful work life are quickly at risk. Male students particularly have a high risk for low achievement, truancy, or delinquency (e.g., Hascher and Hagenauer, 2010). These often lead to a complete abandonment of school education (Harber, 2002; Hascher and Hadjar, 2018; e.g., Havik and Ingul, 2021).

## Context

Contemporary schools struggle to solve and remedy the difficulties caused by societal processes. The school-based teaching profession is increasingly less attractive to young people, with dwindling numbers choosing this demanding field of work. The first author, as a teacher and school principal for 20 years, had variable success in schools supporting and keeping vulnerable youngsters in the system. These youth were mostly male adolescents in the transition to violence, delinquency, or dropping out of school. Today, even after a decade and a half of scientific research and parallel school consulting, he concludes that now is the time for alternatives. Systems operating compulsory education need options, as do other current forms of society, which include work lives beset by these difficulties. Realizing that even a differentiated welfare state system can only absorb the consequences of a capitalist economic system up to a certain point is the core of the problem. Initiating transformative community projects with solid financial resources would provide an unconditional basic income for stakeholders, who along with their own strengths and high motivation can recharge society from within. These projects bring new dynamics with the promise to embrace and create a new quality of social cohesion. The impetus for this idea began when the first author and his family bought an abandoned farm in East Germany, in Anhalt, 1 h southwest of Berlin. Situated in an underdeveloped region, the farm included a barn, a workshop, a stable building, and land. The farm's origins date from 1884, i.e., the Wilhelmine German period, although additions and extensions are from the German Democratic Republic period.

## Key elements

A critical element of this model is gaining a new understanding of an active civil society and the social communities within that assume social responsibility. A second component is redefining the nature and quality of work, including the conceptual framework, with related ideas of income and work–life balance. A third aspect is the role of entrepreneurship and its embedded nature in the development of new social communities that concurrently take recursive responsibility and are based on principles of sustainability. Fourth are the concepts of self-directed and community-oriented learning, which are central components of these entrepreneurial thinking and socially responsible social units. Fifth is the binding element that holds all activities within the project framework together. This component is the idea of working on an overarching economic, social, cultural, and ecological transformation, and seeing oneself as part of such a transformation.

### Rethinking civil society and social communities

Alienation and disintegration in contemporary German society and other modern developed societies can no longer be resolved by state regulation alone. That is, they can no longer be ameliorated by educational institutions and schools, by the welfare state system, or by state support. Therefore, society-wide reforms and renewal initiatives (Lehtola and Ståhle, 2014) and reassessment and redesign of the role of an active, accountable civil society are needed (e.g., Liebert and Trenz, 2009; Wright, 2010; Pérez-Díaz, 2014; Zuk and Zuk, 2022). This examination can be achieved, in part, through discourse analysis related to communitarianism, which critically examines the causes of the crisis in modern societies. A decline in values, loss of solidarity, identity, and meaning are associated with the neoliberal economic and social order. The symptoms of the crisis mentioned are regarded as the consequences of extreme liberalism (e.g., Taylor, 1989, 2012; Sandel, 2008; Walzer, 2009; MacIntyre, 2014). What is important now is that the individual person once again experiences a social embedding. Communitarianism, unlike collectivist societies, preserves the free development and independent thinking of the individual. It embraces social acceptability and takes other people into account.

### Rethinking work, income, and work–life balance

To reduce alienation, we must rethink the concepts of work (e.g., Gomez-Baggethun, 2022), monthly income, and views of the work–life balance. The goal is redesigning and bringing work and personal life into a healthier relationship (e.g., Guest, 2002; Crompton and Lyonette, 2006; Bowers, 2007; Bhende et al., 2020). Introducing an unconditional basic income for all is a key element for the establishment and further development of transformative community projects. There exists a growing body of literature on this subject worldwide (e.g., McKay, 2001; Pateman, 2004; Standing, 2004; Zelleke, 2005; Birnbaum, 2010; Van Parijs, 2013; Levin-Waldman, 2018; Artner, 2019; Delsen, 2019; Torry, 2019; White, 2019; Smith, 2021). These studies support the idea that basic income enables people to collaborate and share economic, social, and educational tasks. A study conducted in India in 2015 purports that basic income positively affects personal health (Beck et al., 2015). It provides the opportunity for people to work part-time, as freelancers, independently, or in small start-ups, originating from these work-related projects. For example, people may practice the skills of making special furniture or ecological agriculture. Ideally, people with diverse professional backgrounds coalesce, roofers and philosophers, carpenters and educators, electricians and doctors, writers, and farmers. Manual and practical work are equally valued in these projects just as much as intellectual work or the handling of financial and business matters. The roles are no longer juxtaposed, act in tandem, providing mutual care and inner balance. Relaxation and contemplation receive sufficient space and the resulting self-determination and social cohesion in professional and personal life yield benefits for the physical and emotional health of all.

### Entrepreneurship and community-building

Traveling by train from Berlin to Anhalt and then cycling through the small villages to the farm, one passes abandoned farms, small businesses, such as a dairy, and vacant properties, where people are given financial stability through a basic income, could come together to build and develop. A growing base of literature on the topic of creating entrepreneurial communities exists (e.g., Markley et al., 2015; Franklin and Dunkley, 2017; Kennedy, 2021; Roulston, 2021; Biney, 2023). Community-based entrepreneurship is of great social importance and provides opportunities, especially when we are dealing with structurally weak areas (Buratti et al., 2022; Mason, 2022). Several studies out of India support the idea of developing entrepreneurs' survival skills (Deka and Goswami, 2020; Shukla et al., 2022) and adopting entrepreneurship to diverse cultural settings and community needs (Torri, 2010). Additionally, there is literature on the topic of building diverse, democratic, sustainable communities (e.g., Martusewicz et al., 2015) and on the development of urban living labs (Marvin et al., 2018). Several forward-looking models already link basic income with innovation and entrepreneurship (e.g., Yun et al., 2019). Another concept that is important for our conceptual and practical work is that of the *commons*. As early as the Middle Ages, there were pastures used jointly by the smallholders. In various regions worldwide, projects based on the idea of the commons are increasingly emerging (e.g., Bollier and Helfrich, 2012; Baldauf and Gruber, 2016; Kirwan et al., 2016; Gruber and Ngo, 2018). In addition, as with these future-oriented considerations, the current farm project is gradually establishing neighborly structures in the village and systems of mutual help and cooperation. For instance, a village farmer helps to pull out fence posts with his tractor, and in return, this farmer cultivates fields on this property. Depending on the season, he shares fresh vegetables or a neighboring family receives larger amounts of firewood for their support services on the farm.

### Self-directed and community-oriented learning

Young people have only been involved in the project temporarily, but let us say that in 10 years, compulsory schooling in Germany changes into a self-designed compulsory affair. Youth could live and learn on the farm in Anhalt for longer. In such a transformed setting, they could mature working for jointly developed goals and social community values. They would live among real people, men and women, craftspeople, and academics, young and old, surrounded by animals, working with tools, experiencing nature, learning by doing, and learning cooperatively. Income stability would ensure that adults are consistently involved in a project, reduce external work, and provide adults on-site who can care for and supervise children. They would have action-oriented learning spaces and dependable adult reference persons and caregivers. Such a pedagogy contains the concepts of experiential education, self-directed learning, and learning responsibility in a social community context, like what David Weikart practiced in the summer workshops which he ran in Michigan for decades, incorporating ideas from Kurt Hahn and John Dewey (Broecher, 2015). Or let us look at the German–Polish exchange pedagogy, geared toward the ideals of international scouting, which Andrzej Jakzewski and his German cooperation partners developed at the time of the Cold War and Iron Curtain (Toczyski et al., 2022). Children grow up in the original projects into which they are born, within a stable system of adult caregivers. This context extends parental reach to represent supplementary role models. Particularly active are fathers and men, a widely underrepresented group in present educational institutions. Their absence challenges the mental and emotional development of many adolescents. Young people would learn about these transformed settings by investigating and applying for a myriad of projects. For example, in Germany, and gradually elsewhere, thousands of such projects would exist, with different profiles, documented on internet platforms so that young people could investigate them and apply for them there. They could, for example, move from a farm with a core profile on ecological livestock farming in Brandenburg to a mill in Lusatia where furniture is made, then to an urban project in Berlin where clothes are designed and tailored, where music is produced, or where jams and juices are made from organically grown fruit in a place where philosophy seminars are also taking place. Adolescence would be self-determined years of journeying. The youth remain in the project for as long as they can learn new things there, and then they move on. Such changes reimagine the concepts of apprenticeship and craft conveyance (Sennett, 2009; Patchett, 2017) and connect them with creativity, innovation, and entrepreneurship education (e.g., Shu et al., 2020).

### Economic, social, cultural, and ecological transformation

**Figure 1:**
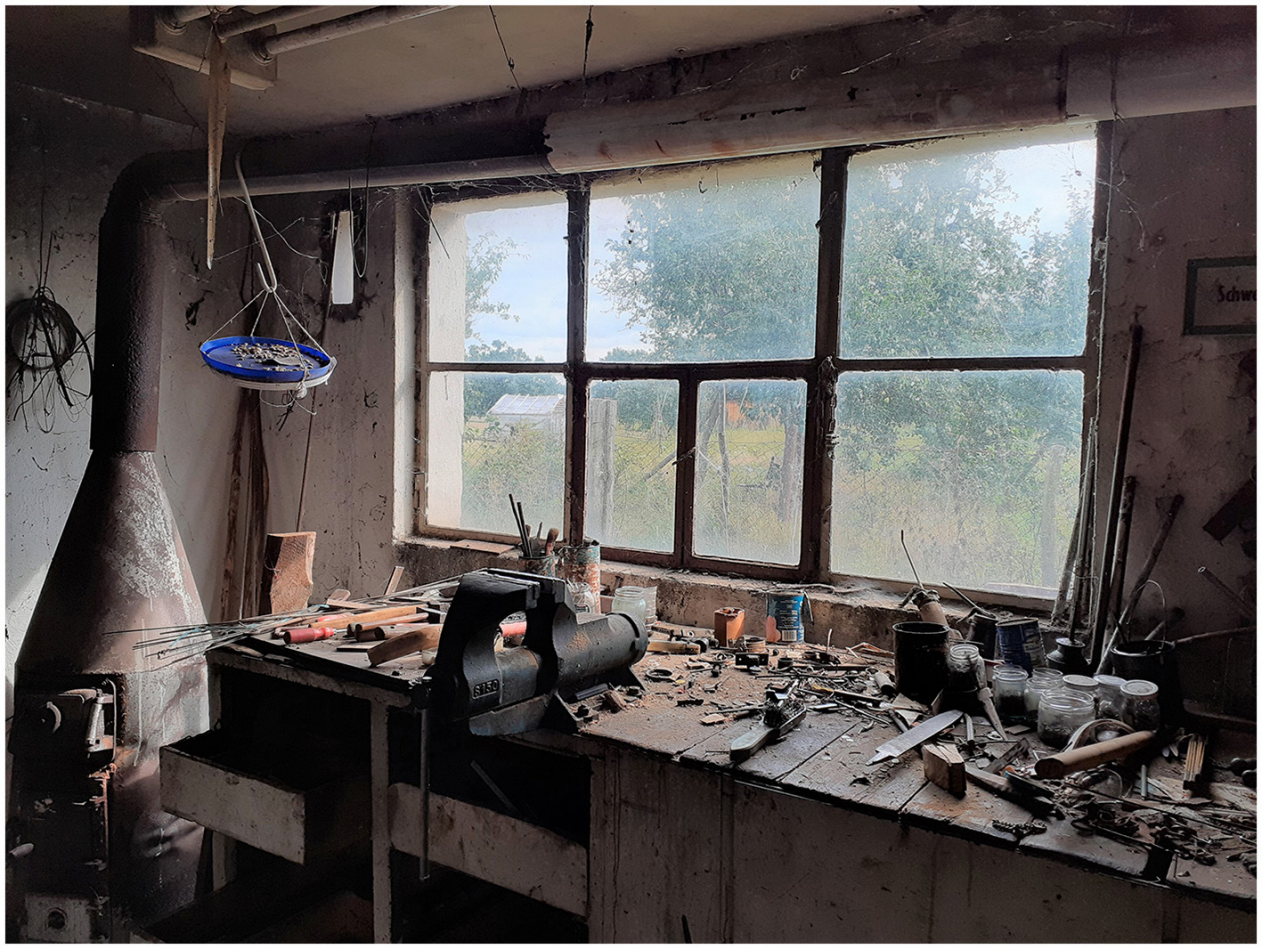
The photo shows the condition of the workshop at the end of 2020. Cleaned and redesigned, discussions are happening about using this workshop in unique novel ways. Smart technologies will be important but also the linking of old manual techniques with new forms of production.

**Figure 2:**
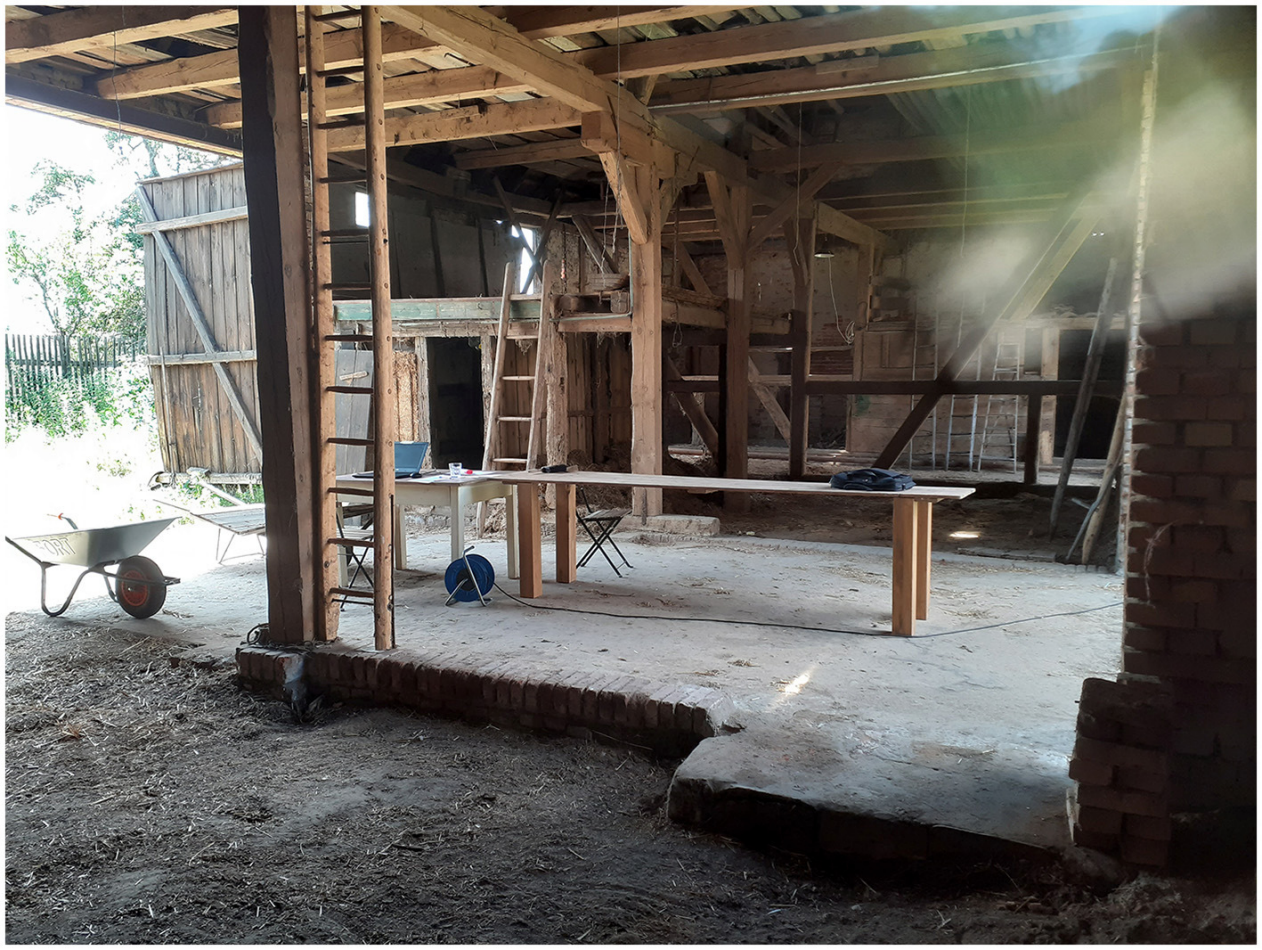
This photo shows the barn interior where the previous contents were removed. The spacious barn could host craft activities, artistic, educational, and cultural activities. For example, in this space, there was a discussion about emotional and social geographies in Polish literature. There was also a meeting with villagers here to discuss the project and its goals.

Inclusive in terms of gender, disability, age, culture, tradition, religion, or language, these transformative community projects reflect a philosophy of belonging and social connectedness, such as that developed by O'Donohue (1997, 1998). The appreciation of the inner richness that each human being brings with him/her seems a helpful reference at present. Inclusive in these projects would be elders. As a result, expensive elder care facilities could be downsized, and the shortage of skilled workers counteracted. Children living and learning in these projects could relieve preschool facilities and schools, which increasingly suffer staff shortages. Increased contact among generations could foster mutual learning, as was once the case in a natural way. Of course, in the past, there were often power structures, dependencies, peer pressure, or a lack of acceptance of individuality. Taking care not to revert to prior times, yet preserving the best ideas and experiences, we would combine them with a new transformative philosophy. The humane values are always decisive, the orientation toward the good, as we can learn from communitarianism or the philosophy of O'Donohue (1998). Along with these social considerations, the farm project in Anhalt is about very practical things (Broecher, 2023a,b). Learning opportunities include repairing, rebuilding, and modernizing historic buildings, farmhouses, stables and barns, and partly timber-framed buildings. These activities preserve the area's cultural heritage and start to implement ecologically valuable building materials and smart technologies (Figures 1, 2). Other learning areas under exploration are organic farming, fruit growing, and sheep farming.

## Discussion

This study aims to connect the critical analysis of social structures with an outline of an alternative model for working, learning, and living, a model that aims at sustainable development for all, including nature, wildlife, and the whole planet (United Nations, 2016). Providing an unconditional basic income and converting state-controlled compulsory schooling into self-designed compulsory education are two central parameters for the change sought here. In short, we advocate the transformation of learning, growing up, working, and living, as areas narrowly intertwined. Significant support exists in German society for establishing unconditional basic income, but there is also opposition. In the United States, many deem this policy as too radical, although there are places open to discussion. Skeptical citizens distrust such a solution, fearing exploitation. Others reject basic income because they see it as a form of social redistribution which encourages passive and selfish behavior. But this project views the presence of a basic income as an incentive to act entrepreneurially and to take on social responsibility. Examining contemporary German society, the conversion of compulsory schooling into a self-designed compulsory education is divisive. Homeschooling and freer learning formats have existed in the United States over time. In Germany, however, opponents repeatedly express a fear that children and young people will resist learning on their own. Conversely, even under the current conditions, there is an emerging group of students who learn too little or who do not go to school regularly, causing a variety of problems, excessive costs, and subsequent problems. Therefore, despite everything, there is a need beyond school for alternative places of learning. This transformative community-project movement, like most reform movements, does not appear without its skeptics and critics. They question the idea of establishing transformative communities because they relate the idea to abuse of power, manipulation, and exploitation. For example, they make the connection with such abysmal projects as *Colonia Dignidad*. To be successful, government agencies must oversee them so that those involved have their human rights guaranteed, and democratic principles are a reality. Transparency and accessibility are paramount to the projects, as are clear philosophical explication and financial disclosure. These projects must prohibit the conditioning or indoctrination of youth in religious, political, and other respects. They must not subjugate ideologies, manipulate, or exploit them. Learning and age-appropriate work conditions should have voluntary participation. It is important to realize that in contemporary German society, there is still a great deal of mistrust of a concept like the commons. This mistrust recalls the socialism that existed, as was present in the GDR and other Eastern European countries. Human character flaws will remain a challenge when, for example, selfishness and egotism prevail in dealing with the resources that should be available to everyone (Hardin, 1968). We hope to stimulate discussion so that people question the fixation on the accumulation of material goods that is dominant in today's Western societies due to capitalism and the manipulation techniques that work within it (Marcuse, 2014), hoping to arrive more at a philosophy of *being* (Fromm, 2013).

## Conclusion

Unconditional basic income would allow for the dismantling of the gigantic administrative apparatuses that distribute social transfer payments. Tax money released could be redirected to the projects, to the people themselves. Such a shift would allow civil society to assume maximum responsibility for itself. The state school system would continue to exist overall, but transformative community projects could reduce their capacity. Young people should never be left with no educational opportunities at all. This approach would provide options for a path to education in a traditional sense, and one to the transformative projects mentioned. Ideally, teacher education of the future could include the context of social, economic, and ecological transformation, including participatory and collaborative practices (Alsop et al., 2007). Thus, the teaching profession could be reinvigorated and charged with new attractiveness. Work would be more self-determined and therefore healthier, resulting in reduced medical expenses. Mobility on the roads could be reduced, with positive effects on the climate, as children and youngsters would no longer have to be shuttled as much, and the sedentary time during transport for adults would be less. This new pace would lead to a decelerated way of life that would benefit people currently living in stressed environments both locally and globally. However, a study from Japan (Klien, 2019) indicated that the mindset of people who long for more self-created work can be strongly influenced by the capitalist system. This study noted that people need to learn to release the mental pressure they carry from such a system. What a fascinating prospect for young people to grow into a healthier world right from the start, with solid social embedding, allowing them to develop their individual potential, always with a view to the overall context on this planet.

## Data availability statement

Publicly available datasets were analyzed in this study. There are two documentation volumes published by Books on Demand, Norderstedt, Germany, about the project with a total of around 700 photos and graphics. Both books are available in print and as e-books. For bibliographic information, see the reference list Broecher (2023a,b).

## Author contributions

Both authors listed have made a substantial, direct, and intellectual contribution to the work and approved it for publication.
