# Supplementary material for: Transformative community projects in East Germany's rural spaces: exploring more sustainable forms of learning, working, and living
Source: Front Sociol. 2023 May 24;8:1164293. doi: 10.3389/fsoc.2023.1164293 (PMC10244620; doi:10.3389/fsoc.2023.1164293)
Supplement: Supplementary file 1 [file Image_1.pdf]

Transformative Community-Projects in East Germany‘s Rural Spaces: Exploring More Sustainable Forms of Learning, Working and Living

Joachim Broecher
University of Flensburg, Flensburg, Germany

Janet F. Painter
Lenoir-Rhyne University, Hickory, NC, United States

Frontiers in Sociology, 8, 2023
Front. Sociol. 8:1164293.doi: 10.3389/fsoc.2023.1164293

Supplementary Material

This file contains additional visuals, with brief descriptions, to give the reader a better idea of what the farm and the project, that is taking place there, is like. The following documentation volumes contain approx. 700 pictures and graphics:

Broecher, J. (2023a). *Tomasz: Notes on Future Ways of Learning, Working, and Living. Educational Projects and Experiences on a Farmstead in Anhalt, Eastern Germany*. Norderstedt: Books on Demand.

Broecher, J. (2023b). *Ludwik: Notes on Future Ways of Learning, Working, and Living. Educational Projects and Experiences on a Farmstead in Anhalt, Eastern Germany, Part II.* Norderstedt: Books on Demand.

In addition, there is the possibility to watch film recordings on the following YouTube channel:

https://www.youtube.com/@otherformsoflearningworkin5830/featured

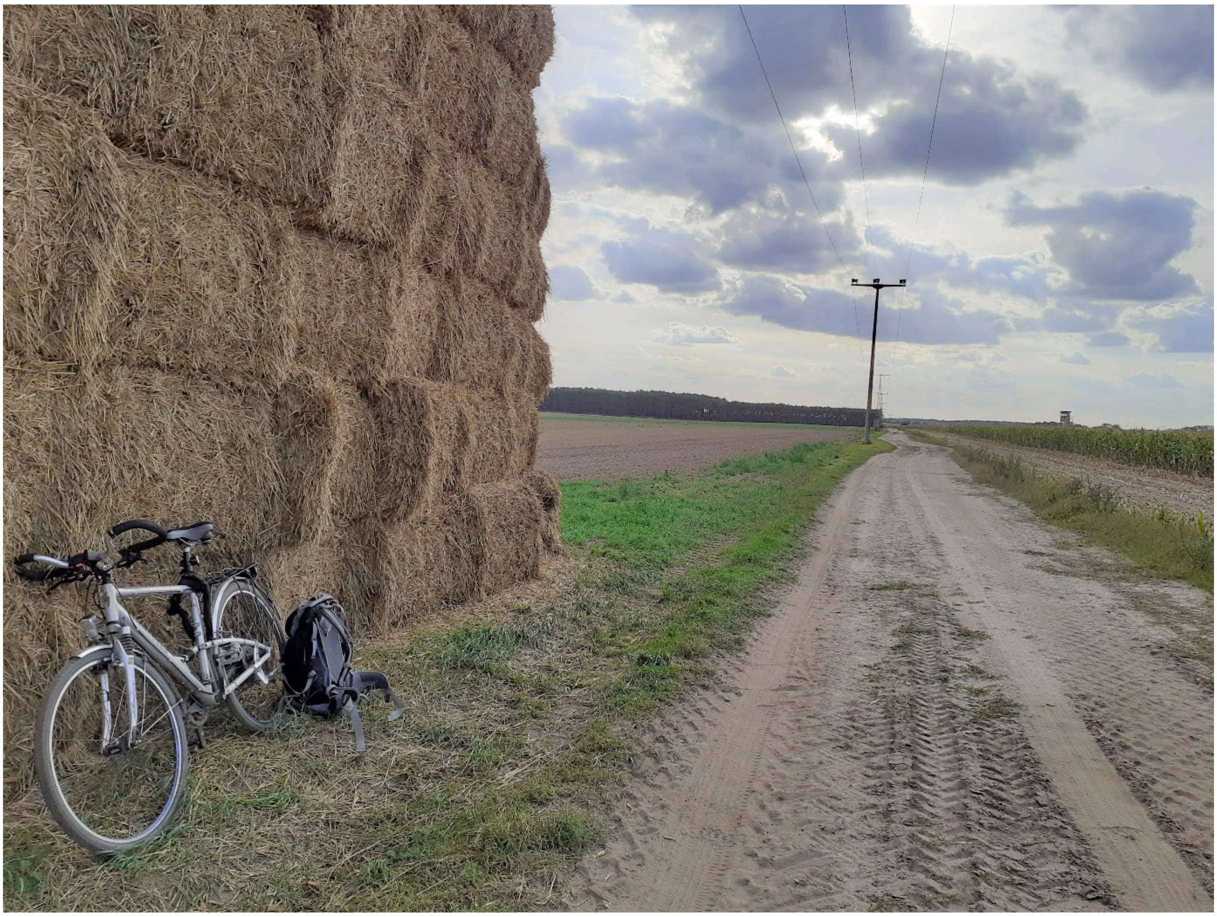

Figure 1: The abandoned train station in Jeber-Bergfrieden, located on the Berlin-Dessau line, seems ripe for a group of people to pool resources, buy it, and develop into a vibrant project. From here, we hop on our bikes and take off down the country road, passing through remote hamlets on the way to our farm.

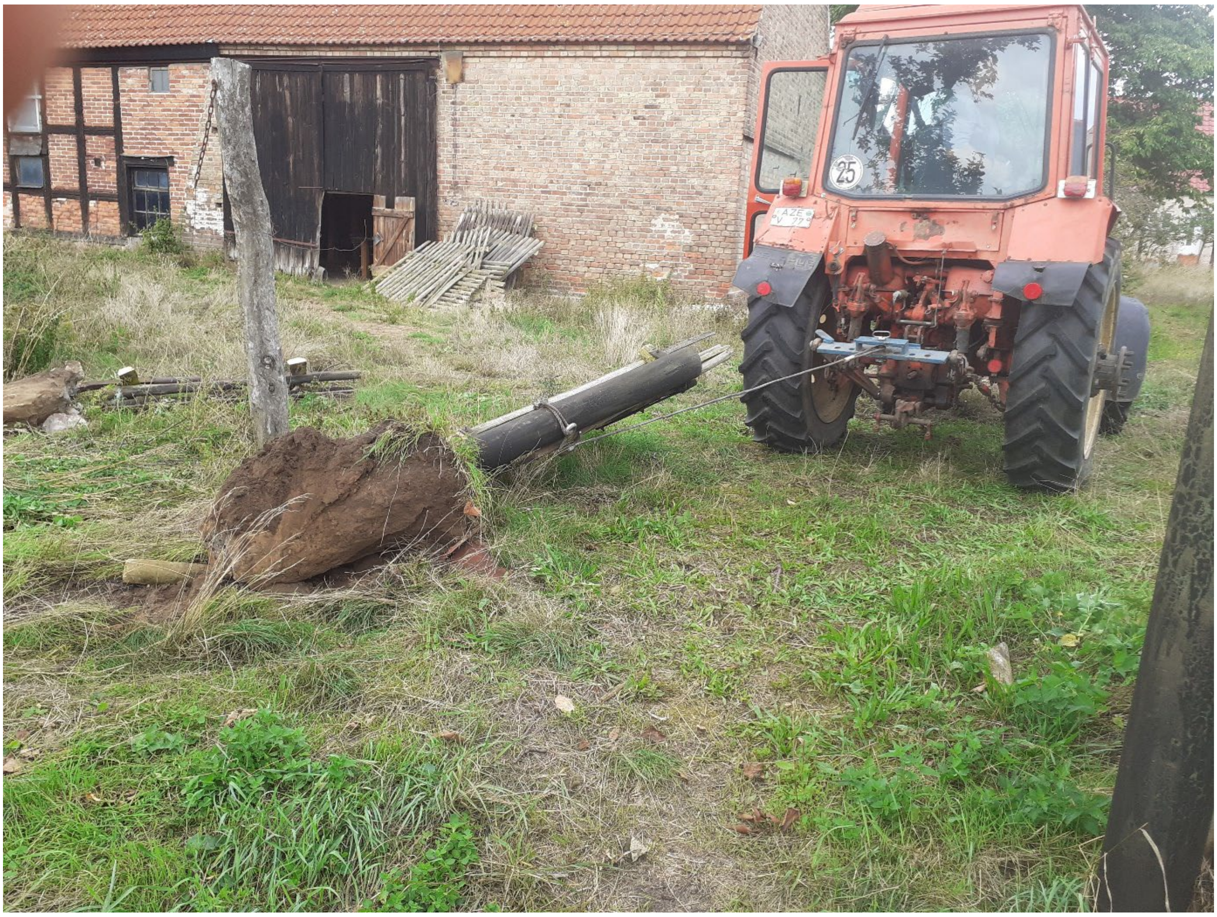

Figure 2: Building structures of help and cooperation among neighbors developed quite naturally. In this picture, one sees a farmer helping to pull out fence posts. In return, this farmer is allowed to cultivate fields on this property as part of his own agricultural production. At the same time, depending on the season, fresh vegetables are shared by him periodically.

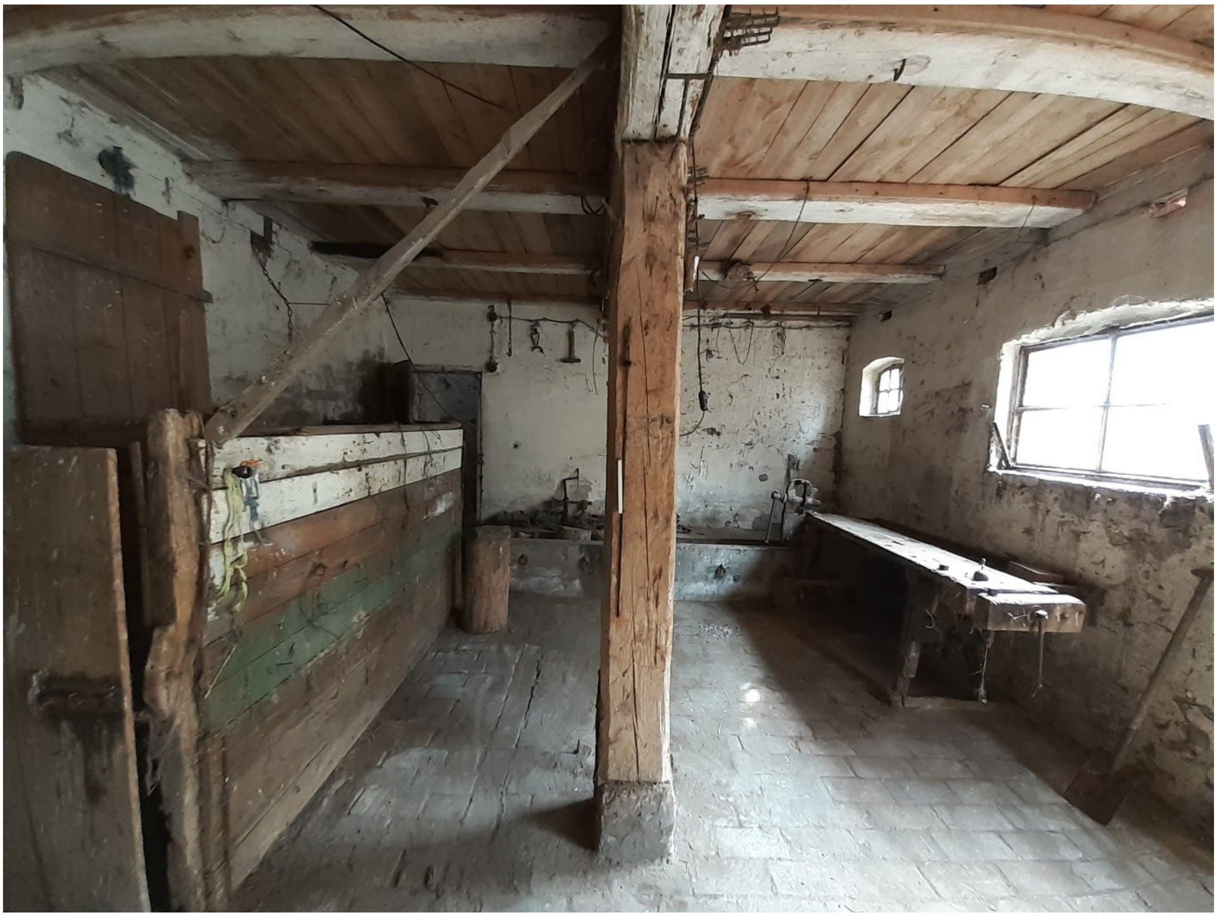

Figure 3: This image includes a part of the former cowshed. Plans have been developed and first steps taken to gut the stable building and convert it into a residential and seminar building.

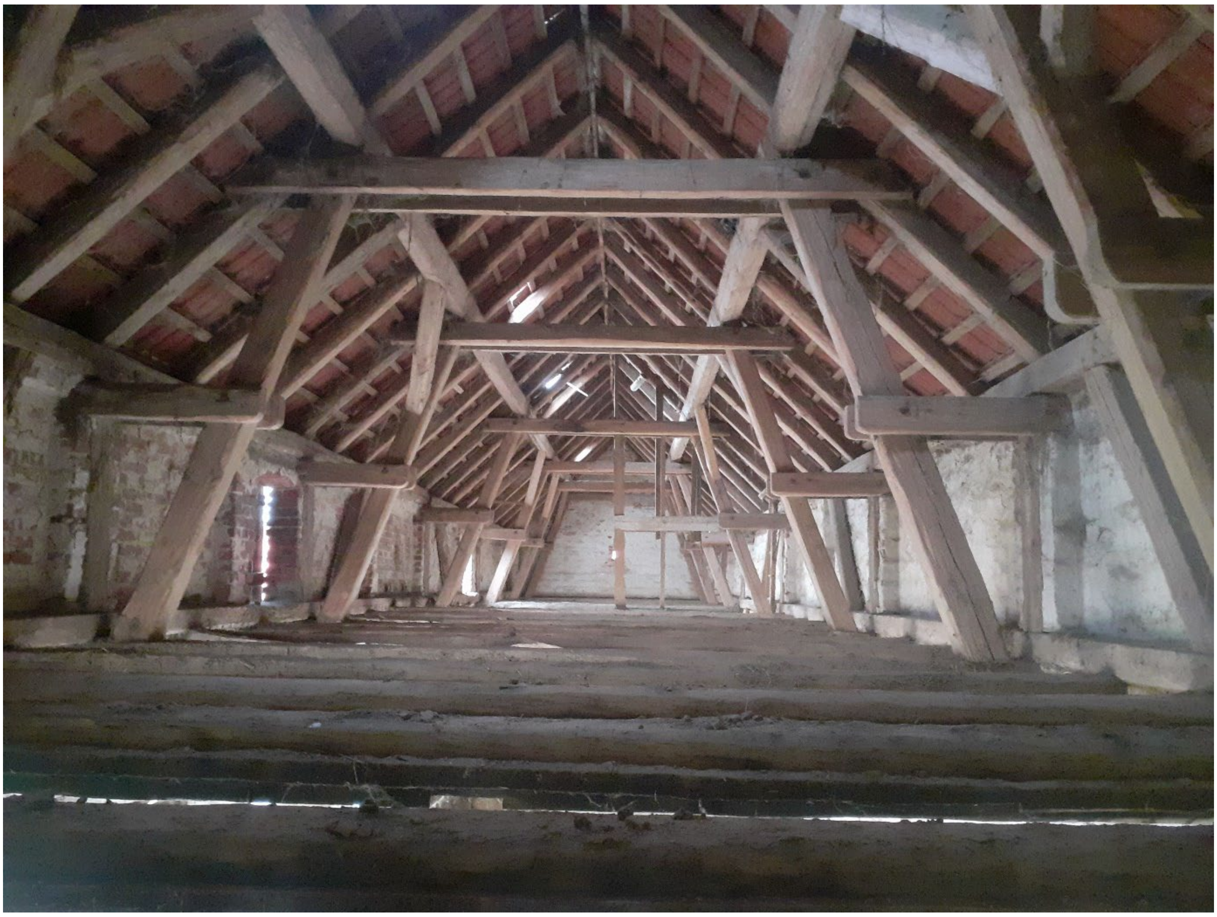

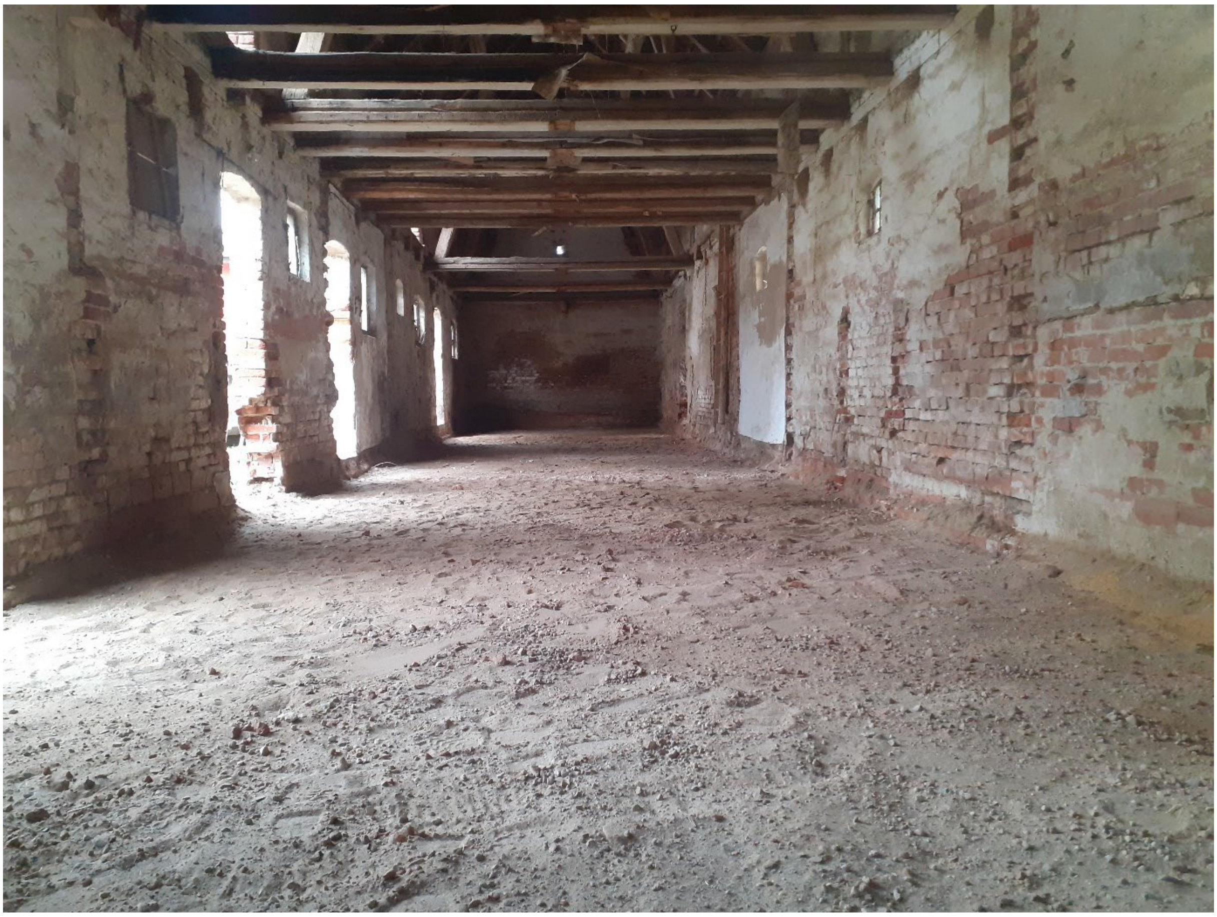

Figures 4-5: The stable building has now been completely gutted.

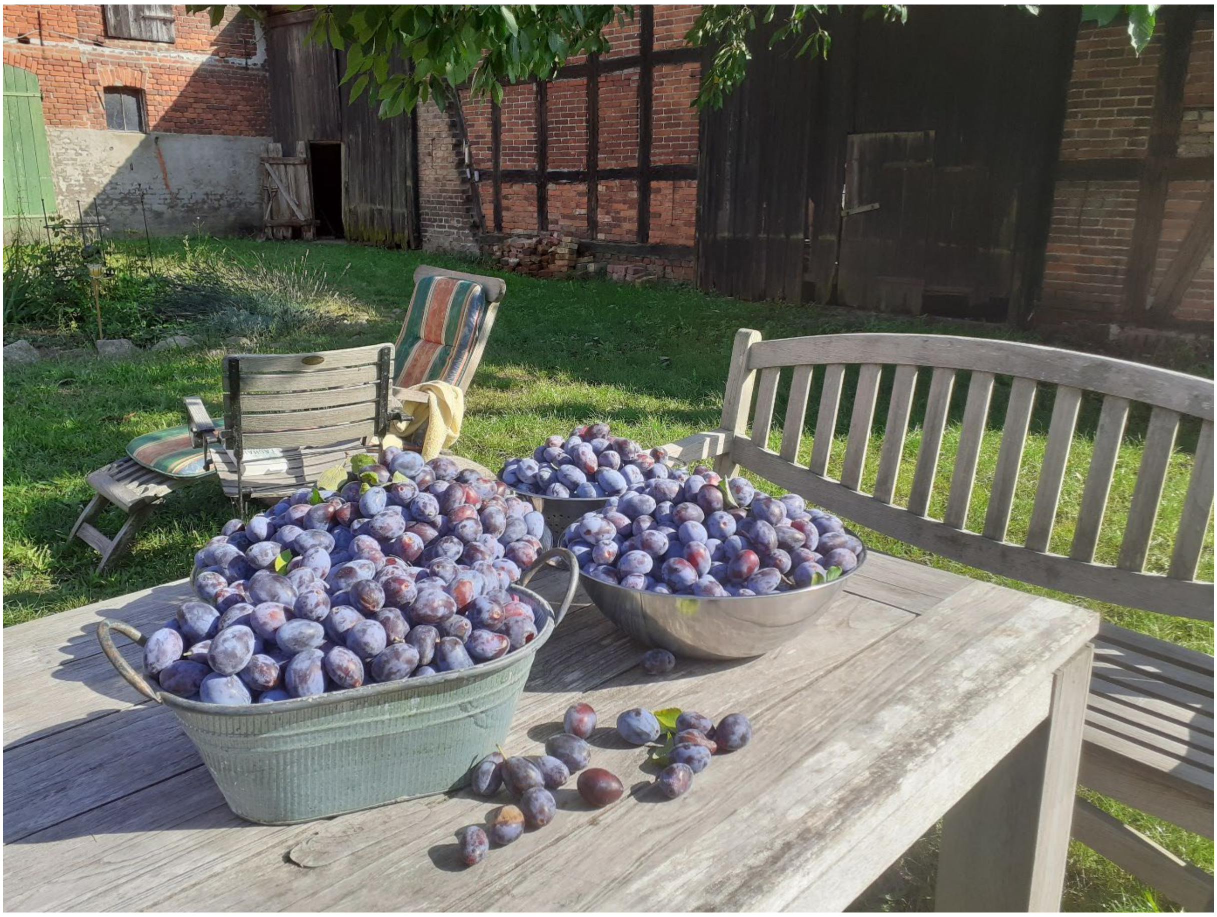

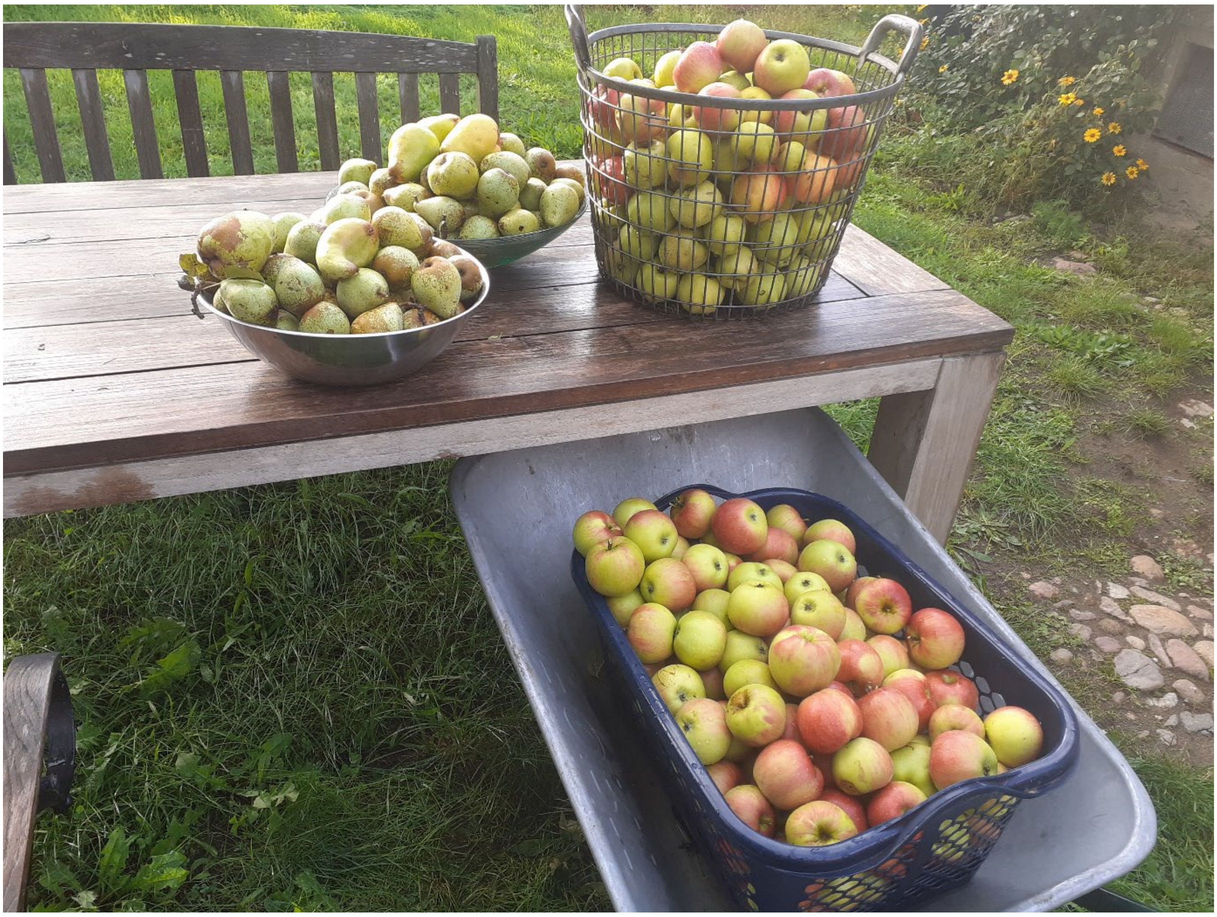

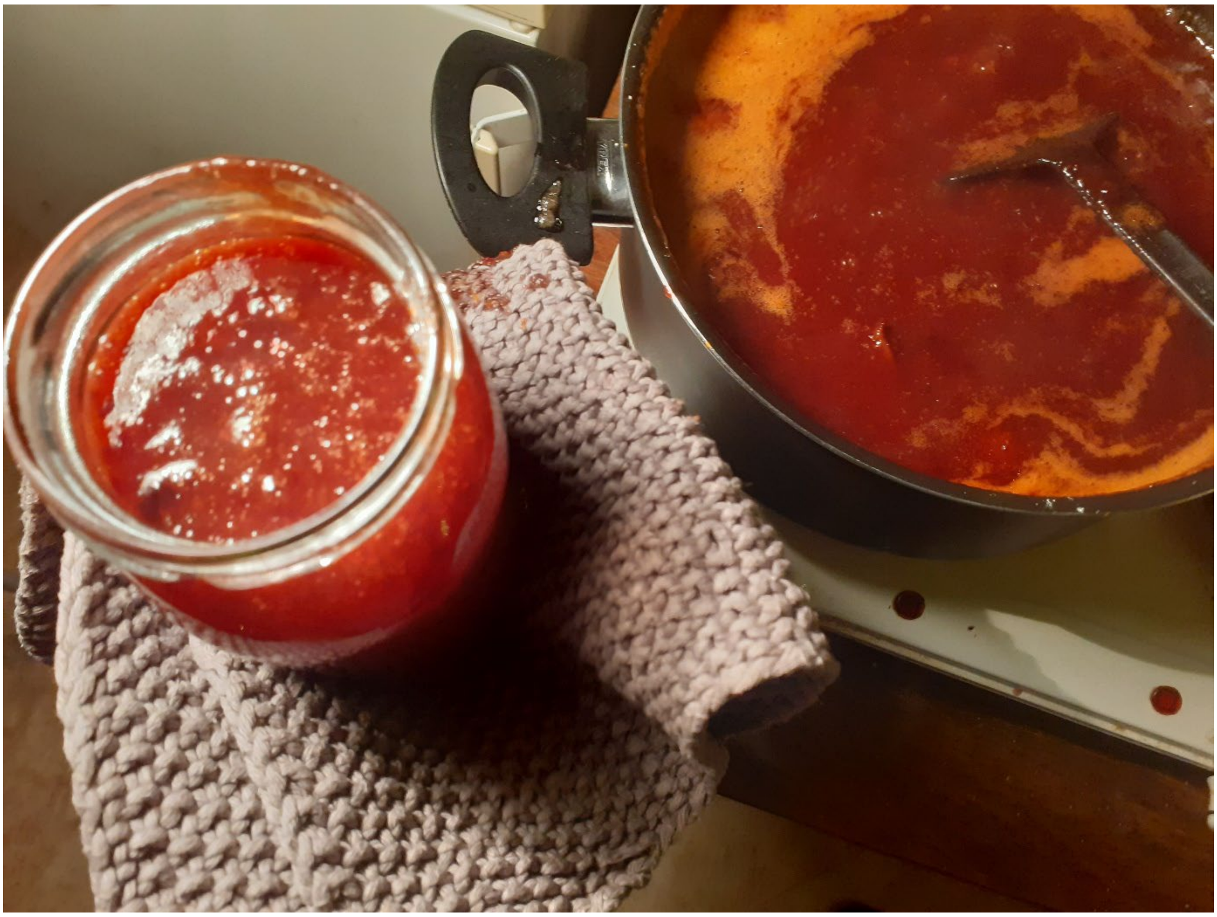

Figures 6-8: Since there are numerous cherry, plum, apple and pear trees on the site, ecological production of jam, fruit cake and fruit juices was started.

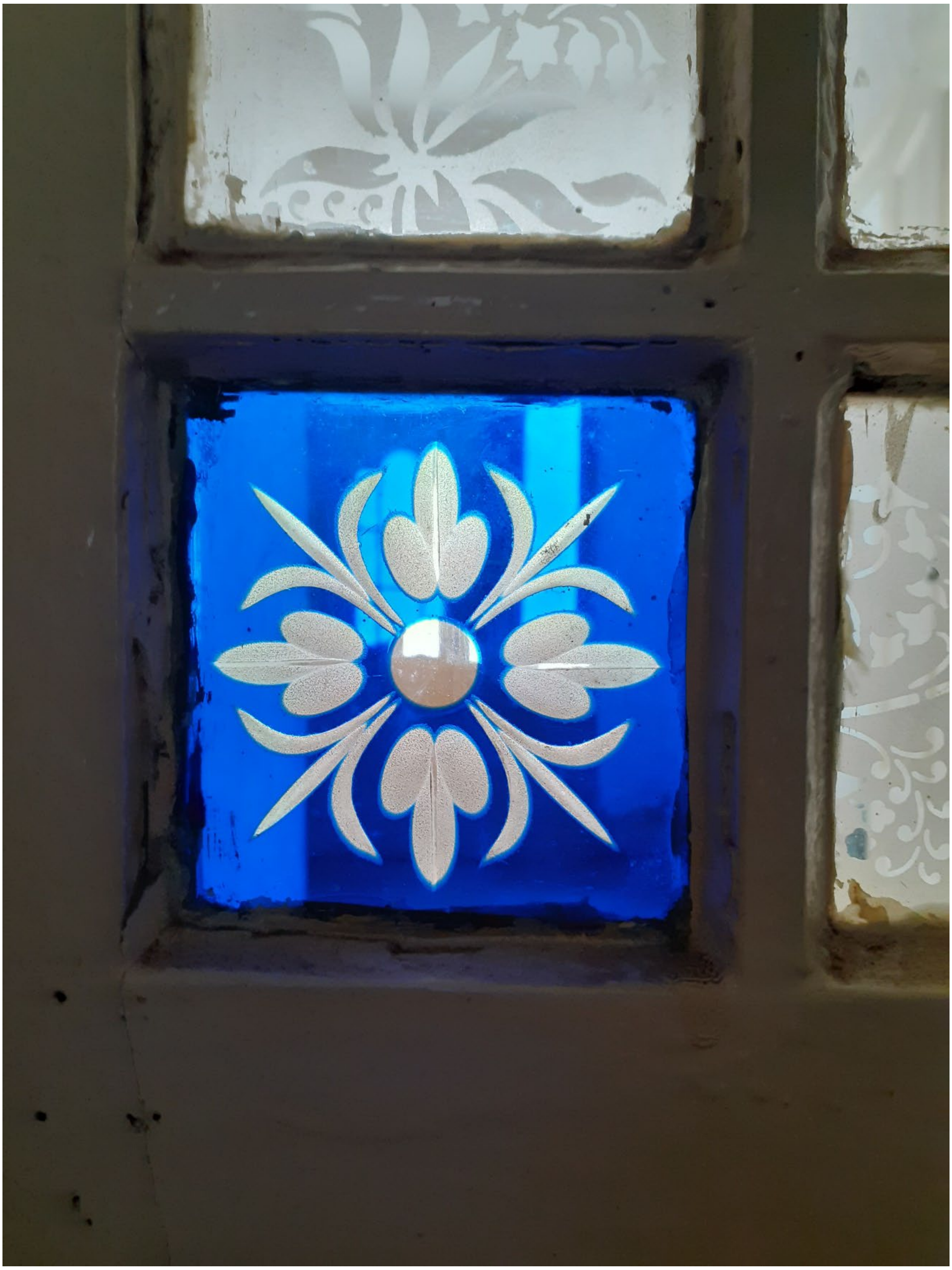

Figure 9: This photo pictures an ornate glass door in the living quarters that dates back to 1884, reflecting the aesthetics of Wilhelmine Germany.

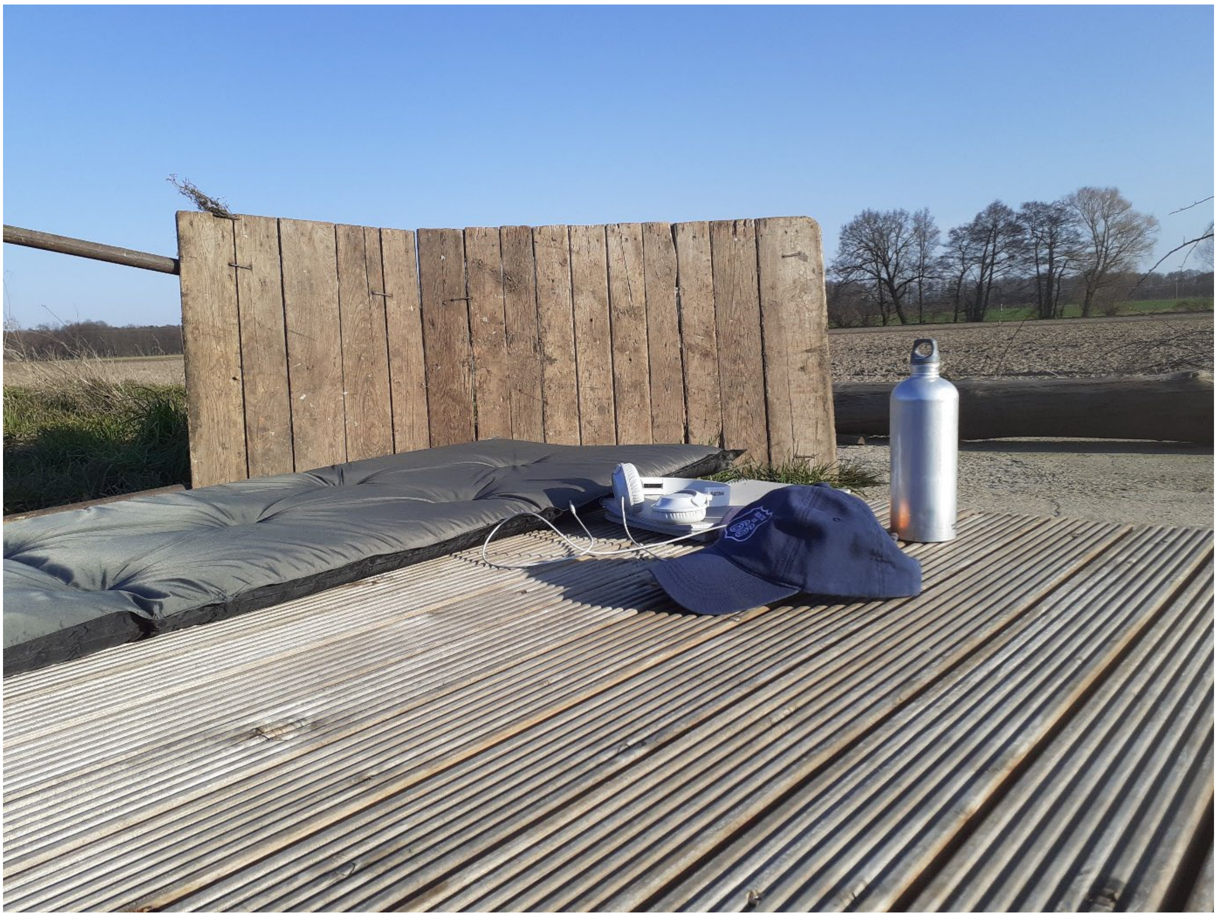

Figure 10: Using a spirit level and small pieces of PVC cut to size, Philipp and Tomasz were able to construct a uniformly level substructure using 2x4s bolted together. They then screwed the wooden surface strips on top of it, spacing them evenly with narrow gaps between them to allow rainwater to drain away. Beforehand, the timbers were coated with red fir oil to weatherproof them. The result: an attractive wooden deck for listening to music, reading, learning languages through a headset, or just gazing at the passing clouds. In winter, we put a brazier of flaming firewood next to the deck, and anyone who wants to stay alfresco just needs to bring a warm sleeping bag.

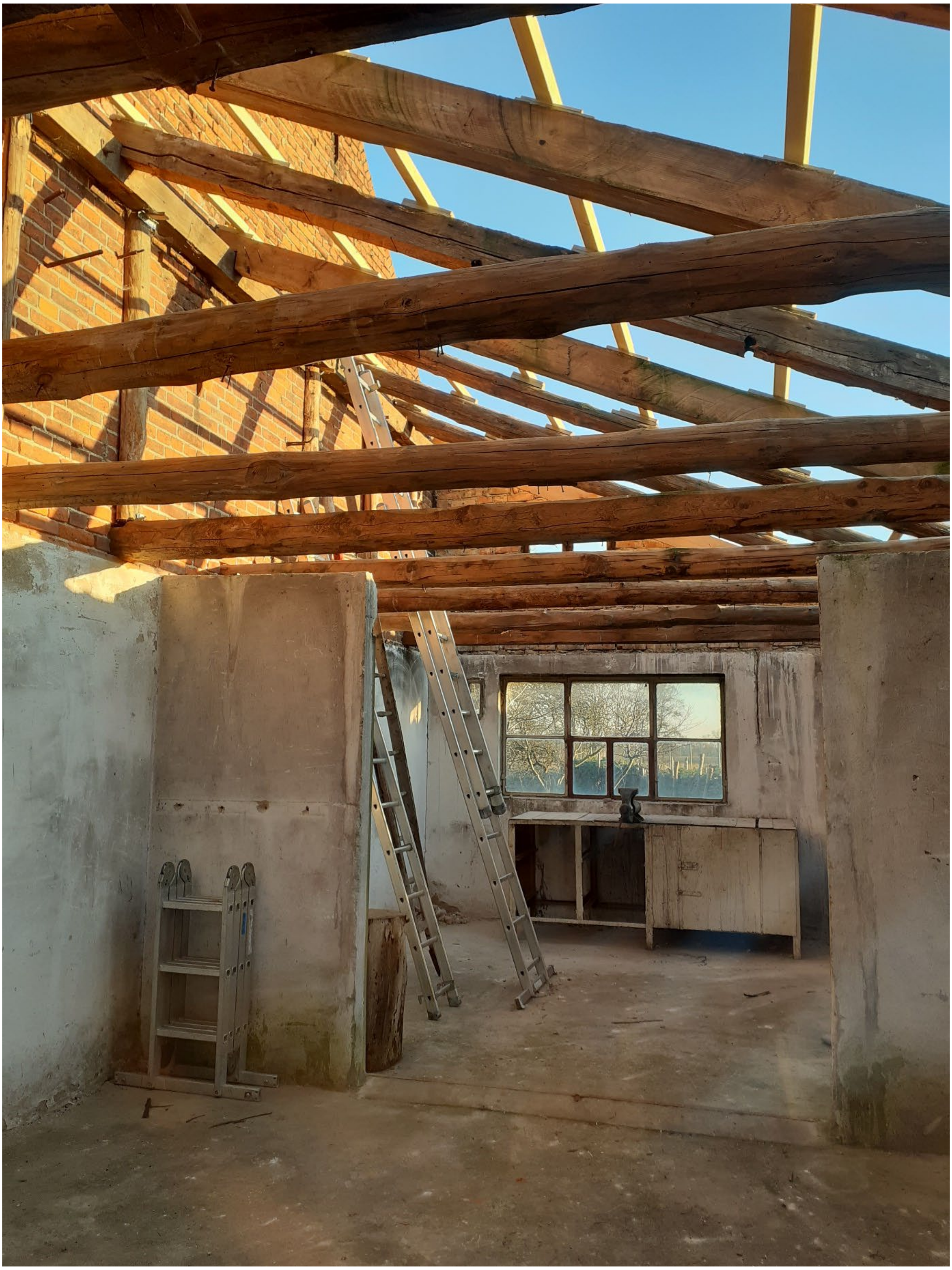

Figure 11: Here we see the workshop, without a roof, without an attic, and all previous items have been removed, except for the anvil and workbench.

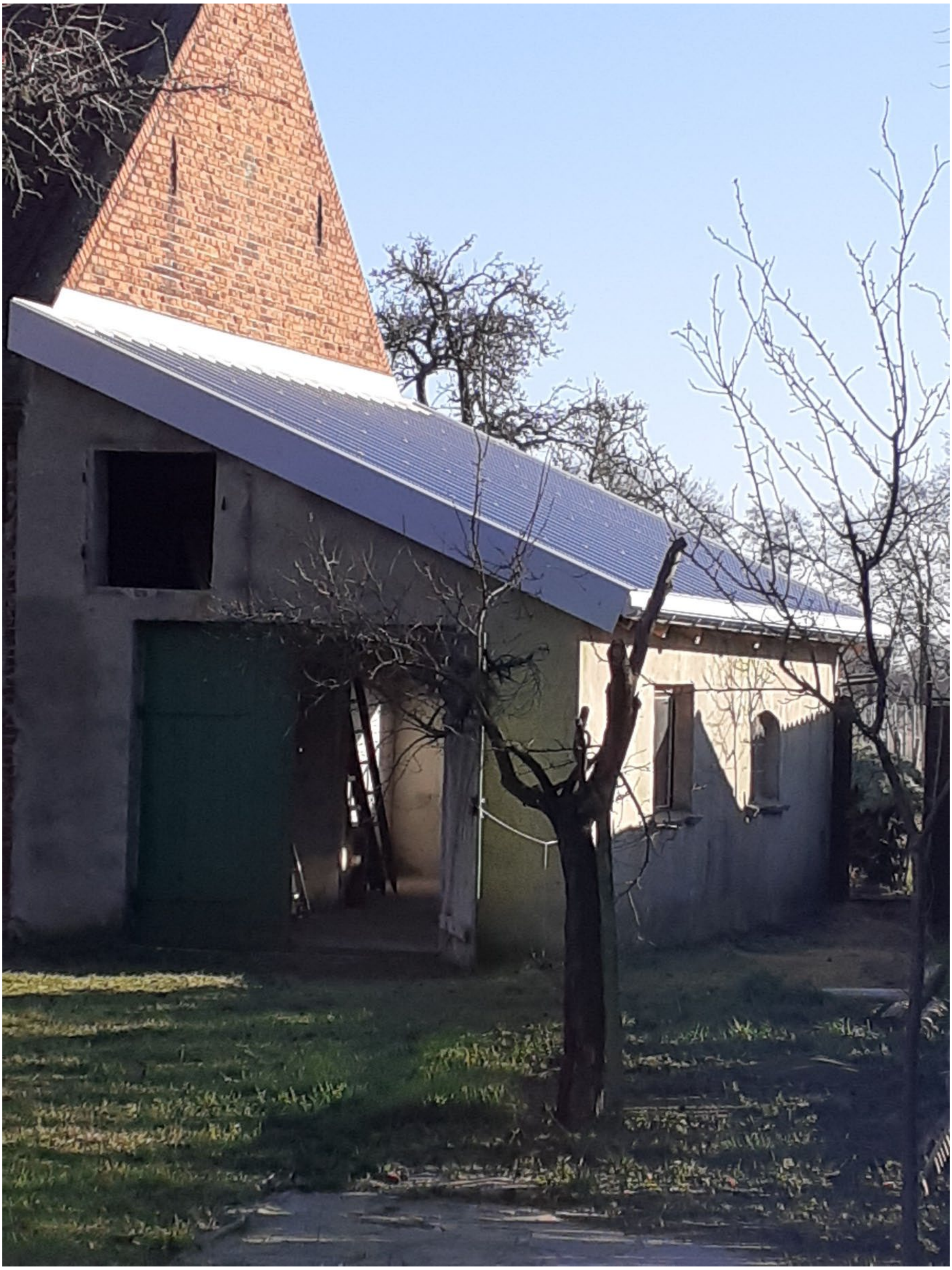

Figure 12: Here the workshop got a new roof, made of high-quality sandwich panels.

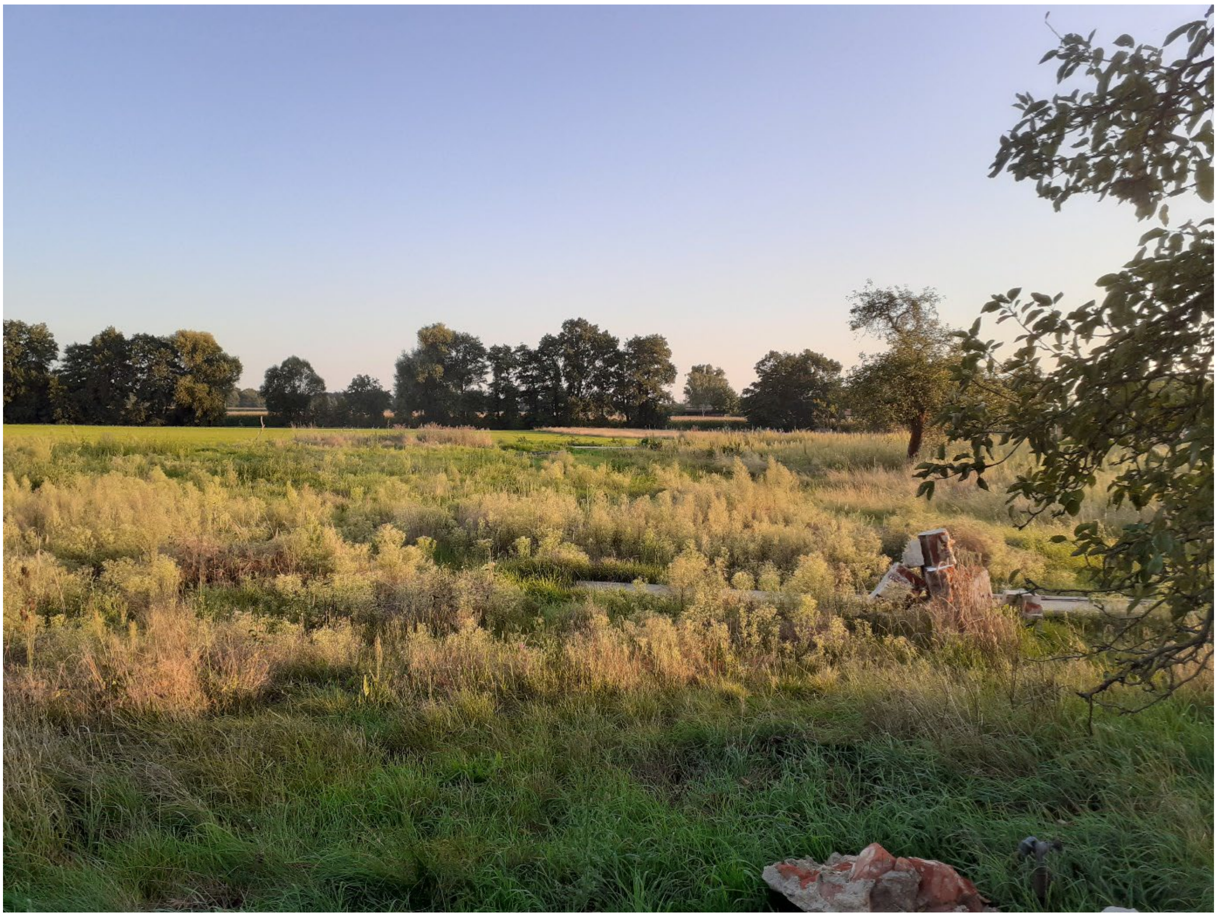

Figure 13: A view of our land behind the barn. We are in a wolf habitat here.

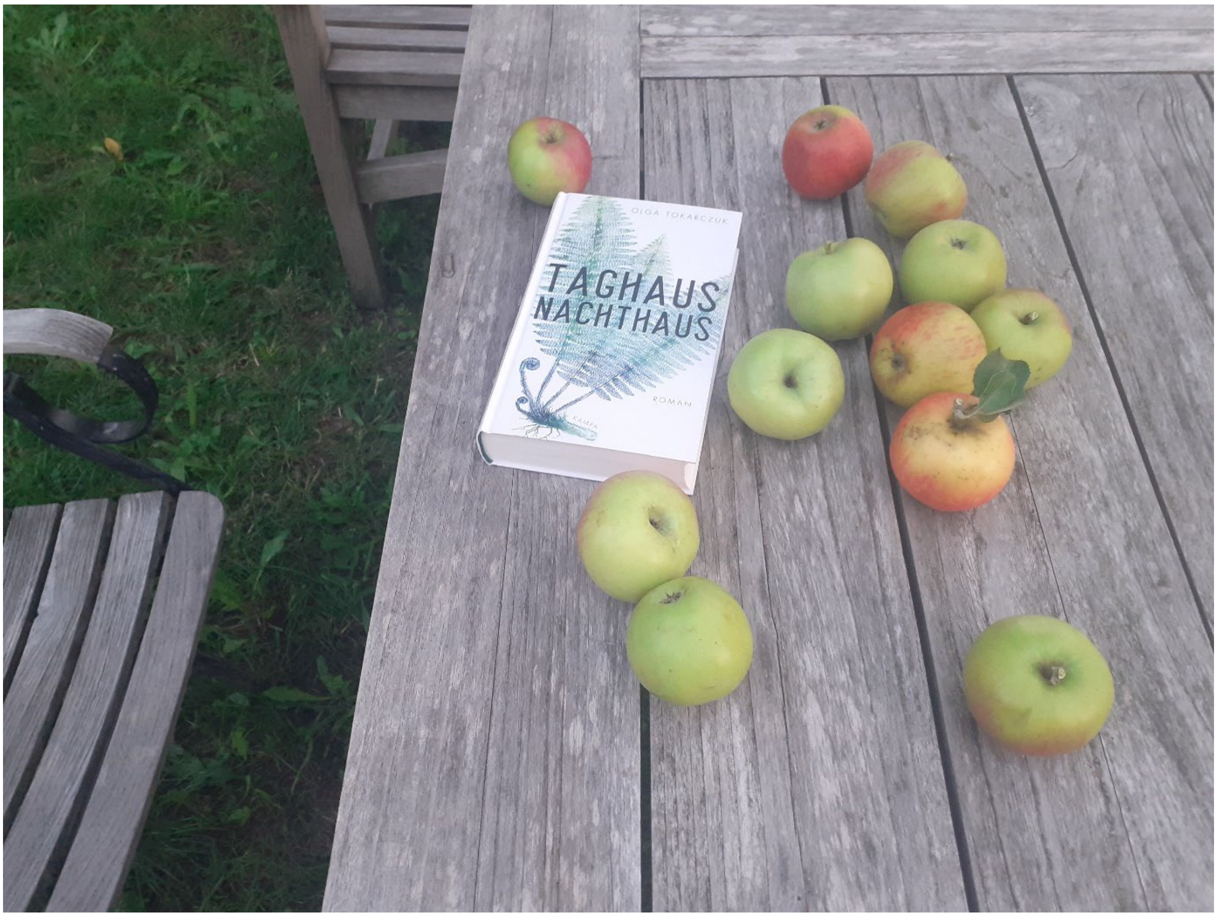

Figure 14: Polish literature in particular plays a central role at the farm as we explore the emotional and social geographies in the works of Polish writers. Especially the books by Olga Tokarczuk are ubiquitous. They open up many perspectives and provide a lot of impetus.

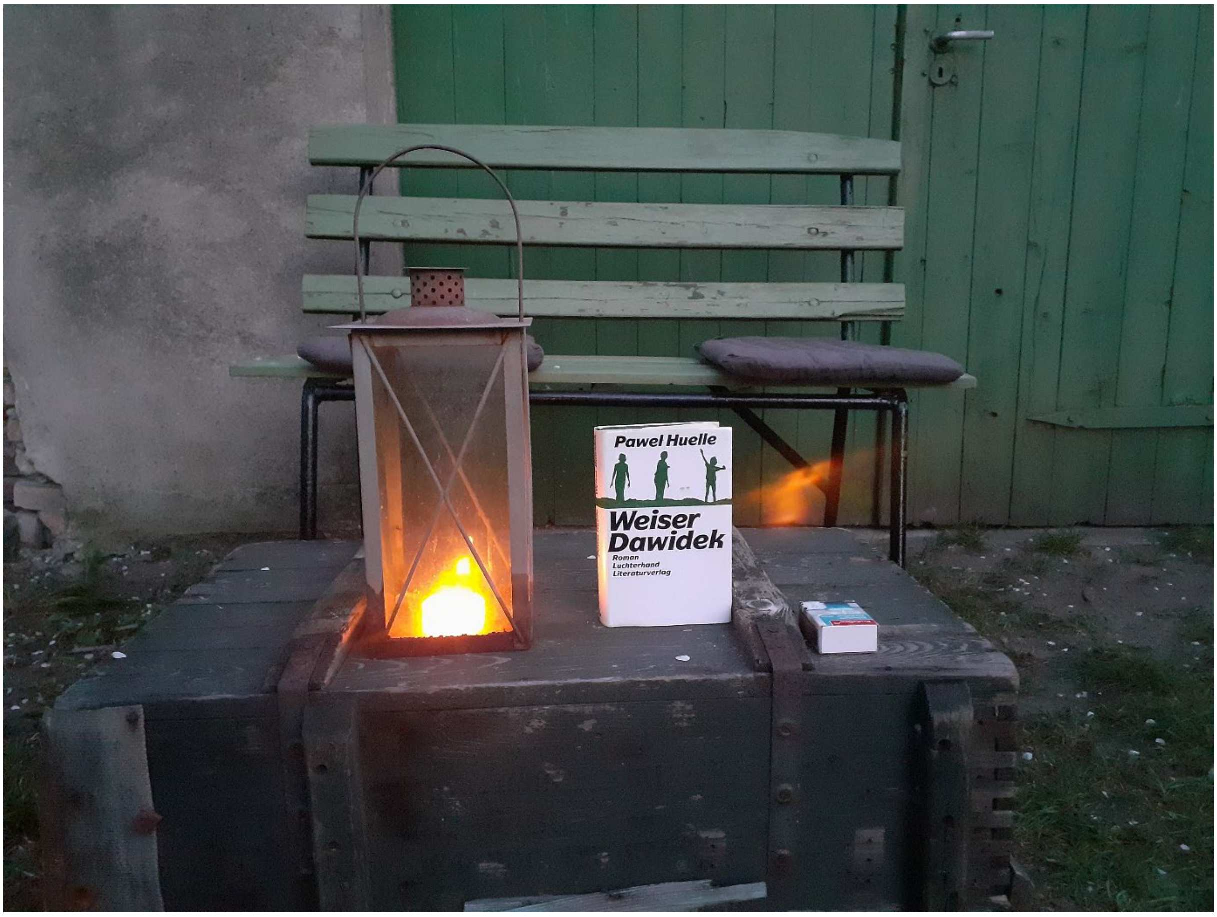

Figure 15: In the evenings, I read Tomasz particularly gripping passages from the books I had previously read myself cover to cover, such as the passage in Paweł Huelle's novel "Weiser Dawidek", a tale of soccer rivalry between groups of boys in postwar Gdansk. Weiser (the protagonist's first name) emerges in the novel as a mysterious figure. For example, Weiser explodes ammunition left over from the Second World War, and he takes on an all-important role in the story. Thanks to Andrzej's pedagogical influence, Tomasz was well versed in classical music, up to and including opera and the works of Stanisław Moniuszko, even some Richard Wagner. Andrzej had always attached immense importance to this connection. He had also exposed this shepherd boy from the Carpathians to historical, social and political topics. In the field of literature, too, the boy already displayed an astonishing breadth of knowledge.

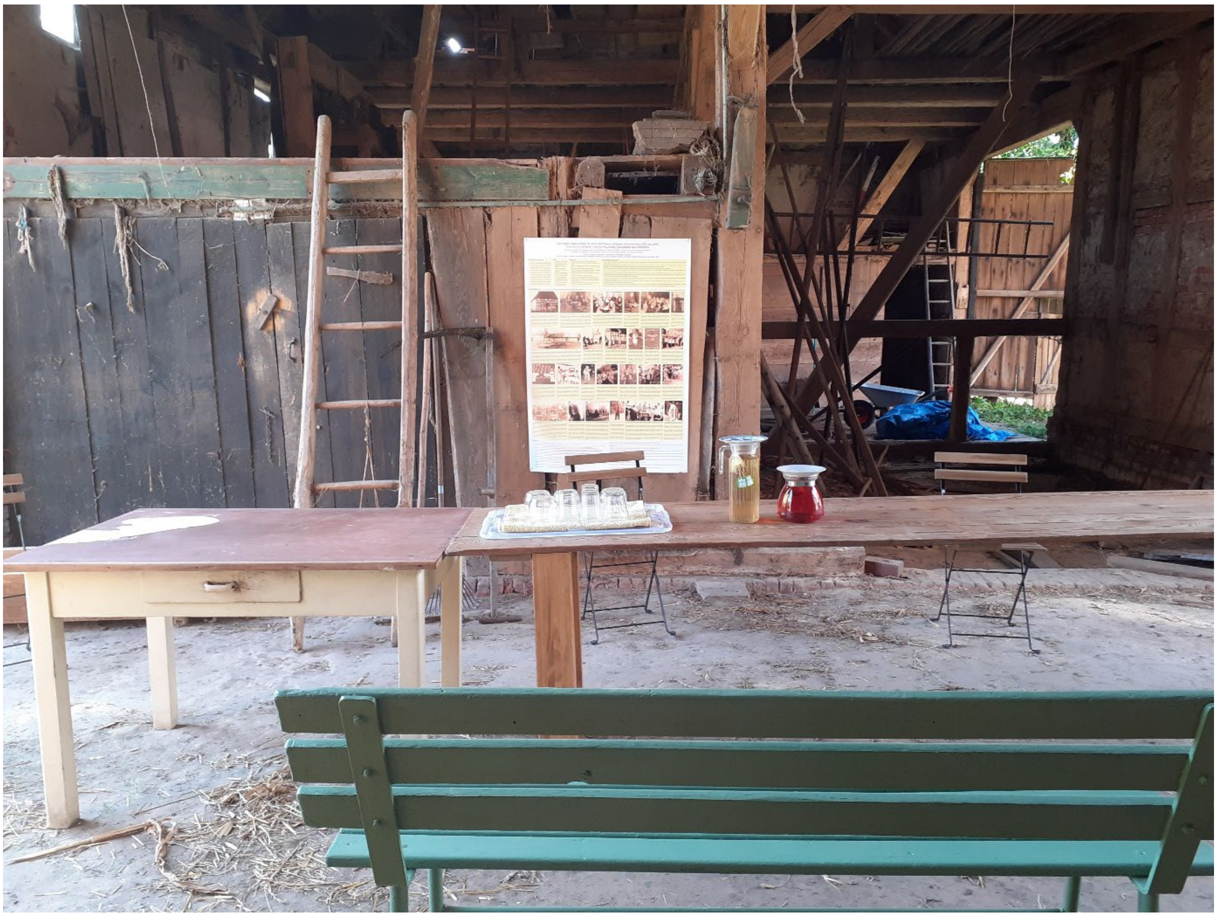

Figure 16: The first reflection processes have already taken place in the barn in the form of informal seminars. The focus was on biographies, generations, and communities of the future.

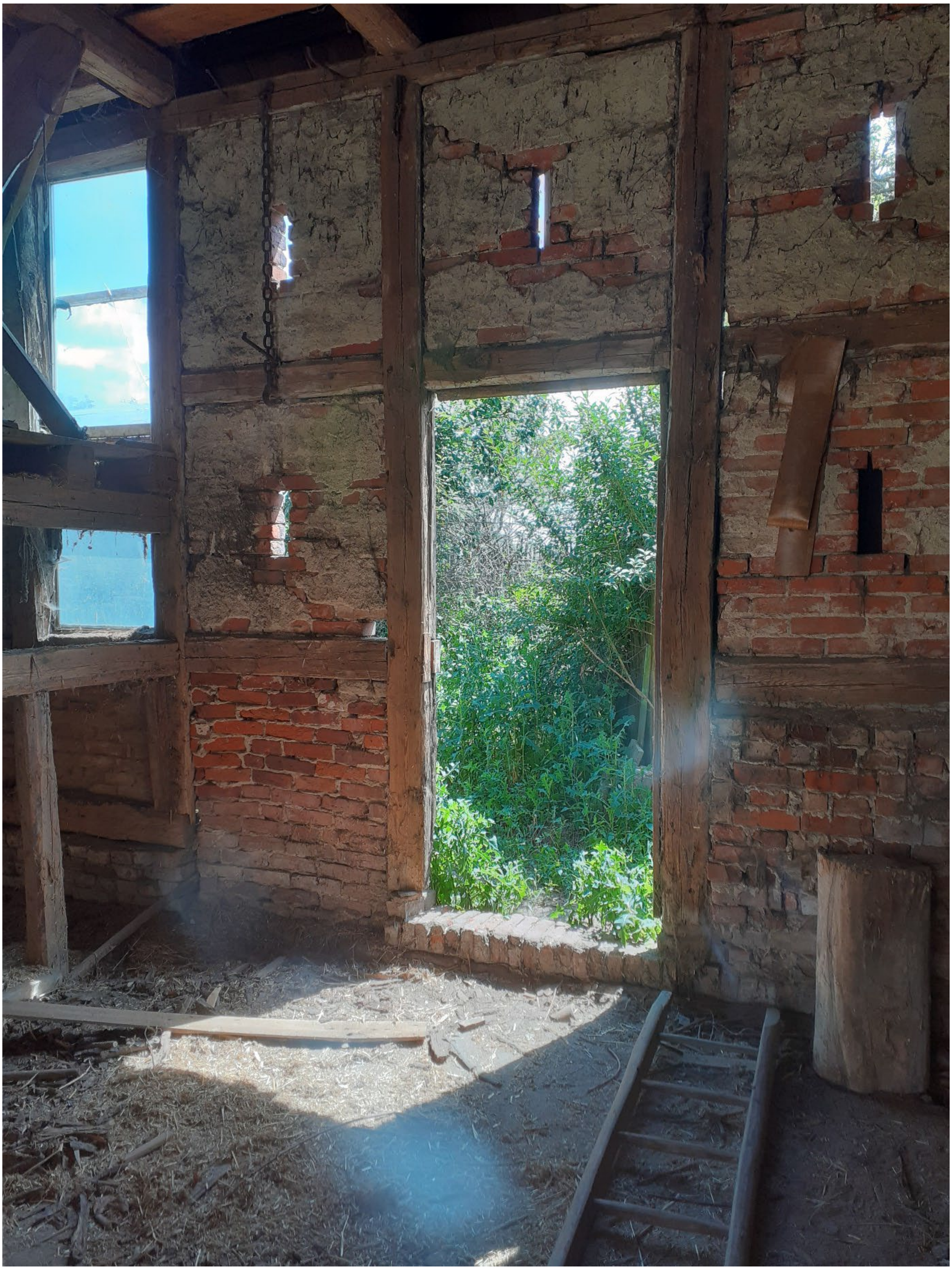

Figure 17: In the writings of the Irish philosopher John O'Donohue, thresholds like this hold the special meaning of consciously entering and exiting from something. They can also denote certain biographical or spiritual developments as part of the creative experience. Depending on how the sunlight falls, it creates a suggestive, almost magical atmosphere by this door. Tomasz often sits here.
